# Why crowding matters in the time of COVID-19 pandemic? - a lesson from the carnival effect on the 2017/2018 influenza epidemic in the Netherlands

**DOI:** 10.1186/s12889-020-09612-6

**Published:** 2020-10-06

**Authors:** LUMC-COVID-19 Research Group, Qingui Chen, Myrthe M. A. Toorop, Mark G. J. de Boer, Frits R. Rosendaal, Willem M. Lijfering

**Affiliations:** 1 Department of Clinical Epidemiology, Leiden University Medical Center, Box 9600, Albinusdreef 2, Leiden, 2300 RC The Netherlands; 2 Department of Infectious Diseases, Leiden University Medical Center, Leiden, The Netherlands

**Keywords:** COVID-19, Influenza, Epidemiology, Mass gathering medicine

## Abstract

**Background:**

To evaluate the association between crowding and transmission of viral respiratory infectious diseases, we investigated the change in transmission patterns of influenza and COVID-19 before and after a mass gathering event (i.e., carnival) in the Netherlands.

**Methods:**

Information on individual hospitalizations related to the 2017/2018 influenza epidemic were accessed from Statistics Netherlands. The influenza cases were stratified between non-carnival and carnival regions. Distributions of influenza cases were plotted with time and compared between regions. A similar investigation in the early outbreak of COVID-19 was also conducted using open data from the Dutch National Institute for Public Health and the Environment.

**Results:**

Baseline characteristics between non-carnival and carnival regions were broadly similar. There were 13,836 influenza-related hospitalizations in the 2017/2018 influenza epidemic, and carnival fell about 1 week before the peak of these hospitalizations. The distributions of new influenza-related hospitalizations per 100,000 inhabitants with time between regions followed the same pattern with a surge of new cases in the carnival region about 1 week after carnival, which did not occur in the non-carnival region. The increase of new cases for COVID-19 in the carnival region exceeded that in the non-carnival region about 1 week after the first case was reported, but these results warrant caution as for COVID-19 there were no cases reported before the carnival and social measures were introduced shortly after carnival.

**Conclusion:**

In this study, a mass gathering event (carnival) was associated with aggravating the spread of viral respiratory infectious diseases.

## Background

Viral respiratory diseases, like influenza, occur in most countries in some years, and in some countries in most years: for many they are common annual events. In epidemic years, 10% or more of a population may be infected with influenza; 50% of infected persons will develop symptoms, and an excess number of deaths will occur [1, 2]. Coronavirus disease 2019 (COVID-19) is an infectious disease caused by severe acute respiratory syndrome coronavirus 2 (SARS-CoV-2) [3], which was first reported in China in December 2019 [4]. It has spread globally and resulted in an ongoing pandemic. As of August 292,020, there were more than 24,770,000 confirmed cases and over 837,000 deaths in about 180 countries/regions according to data from the Coronavirus Resource Center of Johns Hopkins University [5].

Although some details about how COVID-19 is spread are still being determined [6], various measures have been taken to contain the epidemic. The measures advocated and implemented differ between countries, but in general, social distancing is a measure that has been adopted in some form by virtually all countries now. This is based on the knowledge that the virus is primarily spread during close contact and by droplets or aerosols produced when people cough, sneeze or talk [7, 8]. Social distancing has contributed to successfully defeating the severe acute respiratory syndrome (SARS) [9]. This could similarly apply to COVID-19. Social distancing measures, including closing schools and workplaces, restricting travel and cancelling mass gatherings, however, are at the same time affecting almost every aspect of daily life thereby raising concerns about their negative economic and public health implications. In such a dilemma, evidence is needed about social distancing and COVID-19 in order to provide useful information for both the public and the policymaker.

Since COVID-19 is a newly emerging infectious disease, there currently is only limited evidence that social distancing reduces spread of the infection. A cohort study from China compared the effective reproduction number during different periods after the outbreak of COVID-19 and concluded that a series of multifaceted public health interventions was temporally associated with improved control of COVID-19 [10]. Similar results were found in another natural experiment in 149 countries, which concluded physical distancing interventions were associated with reductions in the incidence of COVID-19 [11]. These studies are important to increase our understanding of social distancing, but they included several types of distancing interventions at the same time and could not rule out confounding. These two limitations make it difficult to causally evaluate the potential effect of the intervention.

To provide further evidence on the association between crowding and transmission of viral respiratory infectious diseases, we considered carnival in the Netherlands as a proxy for crowding. Carnival in the Netherlands is a festival that is distinctively regional due to historical reasons, where after the secession from Spain in the sixteenth century the areas south of the great rivers (e.g. Maas, Waal) remained Roman catholic, and the regions to the north protestant. Therefore, it is celebrated mainly in the Southern and Eastern regions of the Netherlands by the local community with an emphasis on role-reversal and the suspension of social norms [12]. Activities during carnival include festive parades and music festivals with many visitors, which creates an environment where keeping an interpersonal distance is difficult. In terms of the proxy for COVID-19, we assumed that seasonal influenza is similar to COVID-19, although the latter might have a slightly longer incubation period and be more contagious [13]. The influenza epidemic in the winter of 2017/2018 lasted the longest of all influenza epidemics in the last two decades in the Netherlands and it has been estimated that 900,000 people had symptomatic influenza and over 16,000 had to be admitted to hospitals [14]. Meanwhile, carnival in 2018 occurred in the Netherlands during the influenza epidemic, which made it feasible to evaluate a potential “carnival effect” on influenza on a nationwide scale. Therefore, this study mainly investigated the change of transmission patterns of influenza before and after the carnival celebrations in the Netherlands in 2018.

## Methods

### Study design and data sources

We conducted a population-based surveillance study on patients who were admitted to hospitals with a diagnosis of influenza in the 2017/2018 influenza epidemic (from the 40th week of 2017 (i.e., 2 October 2017) to the 20th week of 2018 (i.e., 20 May 2018)) in the Netherlands.

Data about influenza-related hospitalizations were accessed from Statistics Netherlands (“Centraal Bureau voor de Statistiek”, CBS). In this nationwide population-based database, personal characteristics at individual level were collected from the Personal Records Database, and data of diagnoses registered with hospital admissions in Dutch hospitals were collected from the National Basic Register of Hospital Care of Dutch Hospital Data which included all general and academic Dutch hospitals and two short-stay categorical hospitals (cancer clinic and eye hospital). All data were gathered and combined at an individual level by CBS to ensure privacy of individuals. The study was approved by the Institutional Review Board of the Leiden University Medical Center for observational studies (Reference number Covid Commission 2020–029).

### Inclusion and exclusion criteria

All hospitalizations with a diagnosis of influenza based on International Classification of Diseases (ICD) 10-codes (J09 and J10) in the study period were included, whereas consecutive influenza related hospital admissions of the same patients during the study period were considered as one hospitalization of interest only, unless the interval between the date of discharge and the date of subsequent hospital admissions was greater than 30 days. Cases of influenza-related hospitalization without municipal information were excluded (accounting for 0.2%). The 3 special municipalities (Dutch Caribbean area) were not included in the study.

### Categorization of carnival region and non-carnival region

**Fig. 1:**
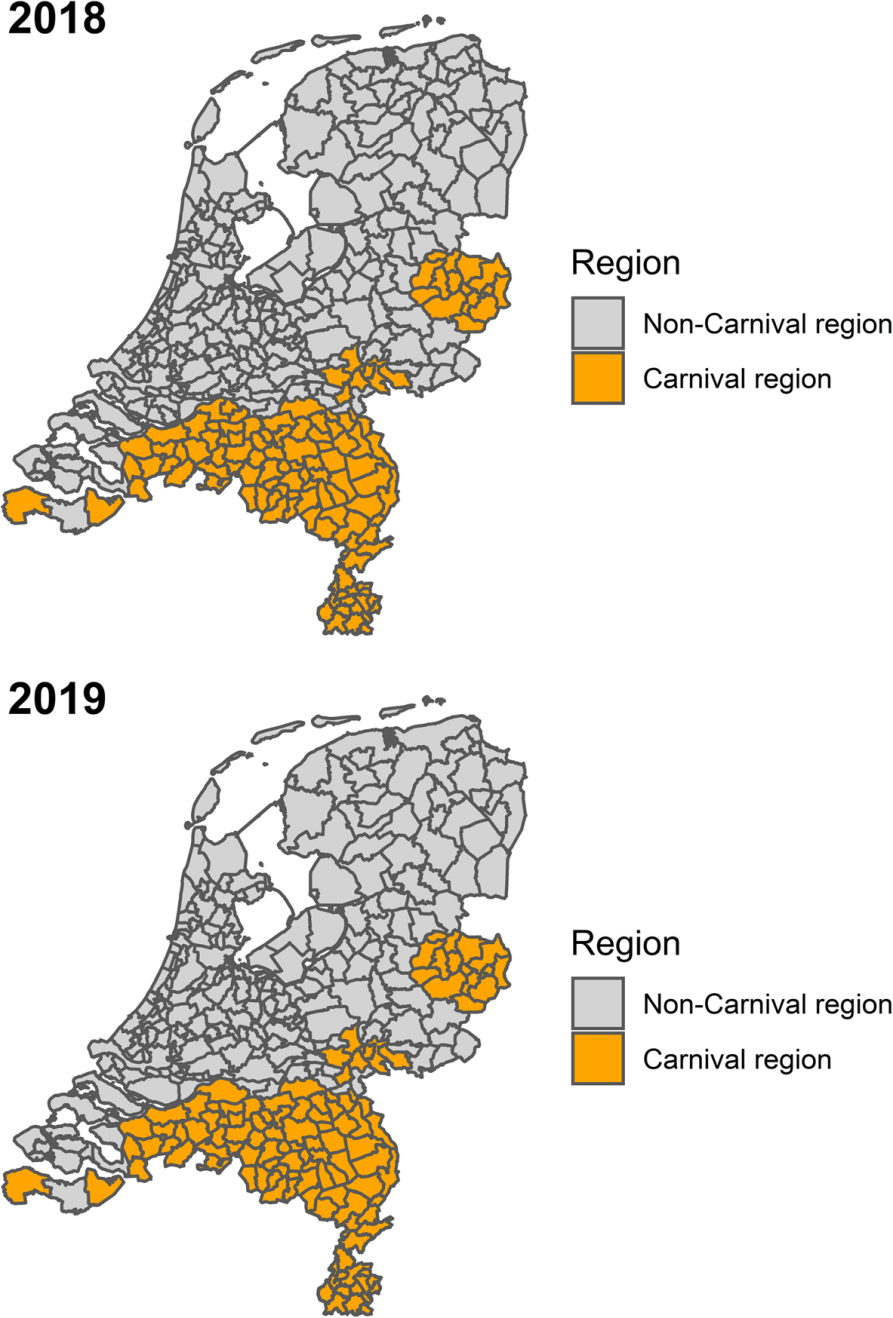
Carnival region and non-carnival region in the Netherlands. Note: The figure was created mainly using packages “cbsodataR” and “sf” with R program (R Core Team (2018). R Foundation for Statistical Computing, Vienna, Austria. Available online at https://www.R-project.org/). The geodata was retrieved via the Application Programming Interface of the Dutch National Georegistry of Public Services On the Map (PDOK) which provides a freely available access to open geo data sets of Dutch governments. Detailed R codes could be found in https://www.cbs.nl/en-gb/our-services/open-data/statline-as-open-data/cartography

The influenza cases were categorized as either from the non-carnival region or from the carnival region based on the municipalities where the patients were registered. The following regions were considered as carnival region: Noord Brabant, Limburg, Twente, the municipalities of Hulst, Sluis, Nijmegen, Over-Betuwe, Lingewaard, De Liemers and Arnhem [15] (Fig. 1, upper panel). The lists of municipalities in the Netherlands in 2018 were accessed from CBS and used to determine the categorization of carnival region and non-carnival region. Detailed categorization at municipal level are presented in Table S1.

### Investigation on COVID-19 cases between regions in the Netherlands

A similar investigation on COVID-19 cases in the Netherlands was also conducted to initially examine potential difference in its transmission pattern between carnival region and non-carnival region. Data on polymerase chain reaction test confirmed COVID-19 diagnoses were accessed from CoronaWatchNL [16] (https://github.com/J535D165/CoronaWatchNL) where daily numbers of COVID-19 cases at municipal level reported by the Dutch National Institute for Public Health and the Environment (RIVM) are collected. Since RIVM stopped reporting COVID-19 cases at municipal level after 30 March 2020, we only included COVID-19 cases diagnosed between 27 February (i.e., the date when the first COVID-19 case was confirmed in the Netherlands) and 30 March 2020. We manually corrected obvious errors probably caused by typographical mistakes in the data (accounting for < 0.3%) before analyses. Similar strategy was used to categorize the COVID-19 cases as either from non-carnival region or carnival region based on the municipalities from which the cases were reported, and cases without municipal information were excluded (accounting for 2.6%). Since which municipality a region belongs to slightly changed over years, the lists of municipalities in the Netherlands in 2019 were accessed from CBS and used to determine the categorization of carnival region and non-carnival region for COVID-19. Detailed categorization at municipal level are presented in Fig. 1 (lower panel) and Table S2.

### Baseline characteristics between carnival region and non-carnival region

Baseline characteristics at municipal level in 2018 and 2019 included the number of inhabitants, population density, degree of urbanity, distribution of sex and age (grouped by 0–15, 15–25, 25–45, 45–65, ≥65 years), marital status, and types of family home (i.e., single-family home referring to any home that also forms a whole building; multiple family home referring to any home that, together with other living spaces or business spaces, forms a whole building). These data were accessed from CBS and gathered based on carnival region and non-carnival region.

### Statistical analysis

Data were expressed as numbers and percentages, or means and standard deviations when applicable. Distributions of new and cumulative influenza-related hospitalizations (or COVID-19 cases) per 100,000 inhabitants were plotted with calendar time (days) in order to compare the transmission patterns between non-carnival and carnival regions. The absolute rate difference and rate ratio in cumulative increase of new cases per 100,000 inhabitants (of influenza-related hospitalization or COVID-19) between regions was calculated, of which the 95% confidence interval (CI) was estimated under the assumption of a Poisson distribution. All statistical analyses were performed with SPSS® Statistics (IBM Corp. Released 2017. IBM SPSS Statistics for Windows, Version 25.0. Armonk, NY: IBM Corp.) and R program (R Core Team (2018). R Foundation for Statistical Computing, Vienna, Austria. Available online at https://www.R-project.org/).

## Results

### Characteristics of inhabitants in non-carnival region and carnival region

The carnival region consisted of 121 municipalities among the 380 municipalities in 2018 with a population of about 4.9 million, while the non-carnival region had a population of 12.3 million. As presented in Table 1, the two regions shared similar characteristics including density of inhabitants, degree of urbanity, mortality rate. These patterns were largely the same in 2019 (Table S3).

**Table 1:** Comparison of regional statistics between non-carnival region and carnival region in 2018

Variables	Non-carnival region	Carnival region
No. of inhabitants	12,310,248	4,870,836
No. inhabitants per km^2a^	437	435
Degree of urbanity^ab^	4	4
No. mortality	107,166 (0.9)	46,197 (0.9)
Sex		
Male	6,095,518 (49.5)	2,431,523 (49.9)
Female	6,214,730 (50.5)	2,439,313 (50.1)
Age groups (year)		
0–15	2,030,940 (16.5)	731,684 (15.0)
15–25	1,517,463 (12.3)	599,350 (12.3)
25–45	3,081,548 (25.0)	1,141,066 (23.4)
45–65	3,424,685 (27.8)	1,415,232 (29.1)
≥ 65	2,255,612 (18.3)	983,504 (20.2)
Marital status		
Unmarried	6,036,871 (49.0)	2,250,736 (46.2)
Married	4,730,875 (38.4)	1,979,300 (40.6)
Separated	951,717 (7.7)	372,909 (7.7)
Widowed	590,785 (4.8)	267,891 (5.5)
Type of family home^a^		
Single family home (%)	80	85
Multiple family home (%)	20	15

^a^ Median of the region

^b^ According to the environmental address density, an urban class has been assigned to every neighborhood, district or municipality. The following class division has been used: 1, very strong urban> = 2500 addresses per km^2^; 2, strongly urban 1500–2500 addresses per km^2^; 3, moderately urban 1000–1500 addresses per km^2^; 4, few urban 500–1000 addresses per km^2^; 5, non-urban < 500 addresses per km^2^

### Distributions of influenza-related hospitalizations with time between regions in 2017/2018 influenza epidemic in the Netherlands

**Fig. 2:**
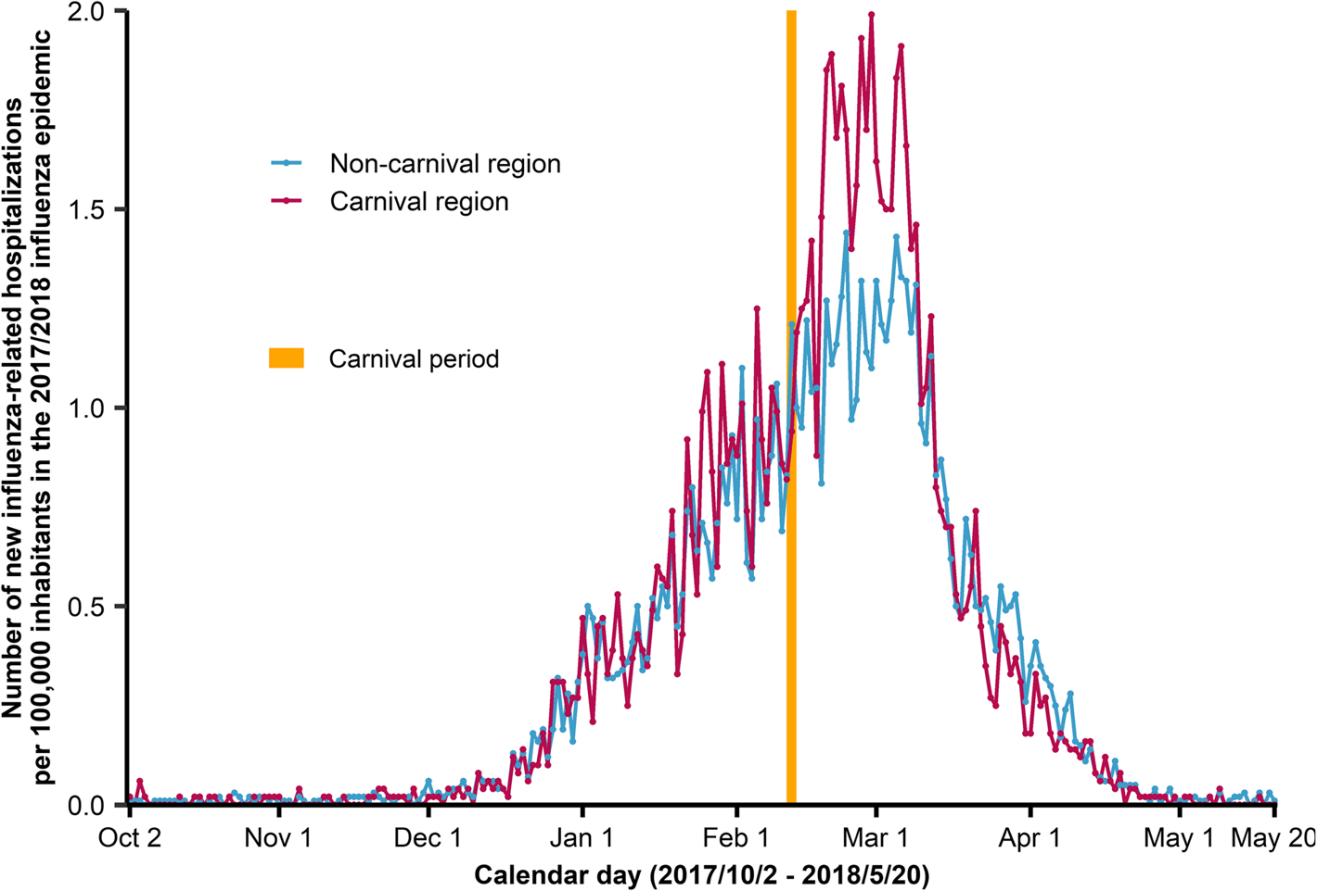
Daily distribution of new influenza-related hospitalizations during the 2017/2018 influenza epidemic in the Netherlands

There were 13,836 influenza-related hospitalizations in total in the 2017/2018 influenza epidemic in the Netherlands. Carnival in 2018 occurred about 1 week before the peak of weekly new influenza-related hospitalizations. The distributions of new influenza-related hospitalizations per 100,000 inhabitants with time between regions followed the same pattern, but slightly diverged just before carnival, with a major divergence in the week after (Fig. 2). The observed difference between regions in the spread of influenza lasted for 2 weeks. When presented as cumulative cases of influenza-related hospitalizations per 100,000 inhabitants (Figure S1), the increase of new cases in the carnival region started to exceed that in the non-carnival region just before carnival. The gap reached a maximum about 1 week after the carnival. This is consistent with results of the calculation of daily increase of influenza cases (per 100,000 inhabitants) (Table 2, Table S4), indicating that the greatest difference in increase of influenza cases between regions could be observed in the first week after carnival.

**Table 2:** Influenza-related hospitalizations per 100,000 inhabitants in the 2017/2018 influenza epidemic in the Netherlands

Day^a^	Cumulative Cases^b^		Absolute rate difference^c^ (95% CI)	Rate ratio^c^ (95% CI)
	Non-carnival region	Carnival region		
2018/01/28	17.94	18.54	0.60 (− 0.81–2.06)	1.03 (0.96–1.12)
2018/01/29	18.79	19.65	0.86 (− 0.60–2.35)	1.05 (0.97–1.13)
2018/01/30	19.55	20.51	0.96 (−0.53–2.48)	1.05 (0.97–1.13)
2018/01/31	20.49	21.43	0.95 (− 0.57–2.50)	1.05 (0.97–1.12)
2018/02/01	21.21	22.32	1.11 (− 0.44–2.69)	1.05 (0.98–1.13)
2018/02/02	22.31	23.32	1.01 (− 0.58–2.63)	1.05 (0.97–1.12)
2018/02/03	22.92	24.06	1.14 (− 0.47–2.78)	1.05 (0.98–1.12)
2018/02/04	23.49	24.66	1.17 (− 0.46–2.83)	1.05 (0.98–1.12)
2018/02/05	24.46	25.91	1.45 (−0.22–3.16)	1.06 (0.99–1.13)
2018/02/06	25.18	26.83	1.65 (−0.04–3.38)	1.07 (1.00–1.14)
2018/02/07	26.03	27.59	1.57 (− 0.15–3.32)	1.06 (0.99–1.13)
2018/02/08	26.90	28.64	1.74 (−0.02–3.53)	1.06 (1.00–1.13)
2018/02/09	27.96	29.63	1.67 (−0.12–3.49)	1.06 (1.00–1.13)
2018/02/10	28.65	30.49	1.84 (0.03–3.68)	1.06 (1.00–1.13)
2018/02/11^d^	29.48	31.31	1.83 (0.00–3.70)	1.06 (1.00–1.13)
2018/02/12^d^	30.69	32.25	1.57 (−0.30–3.46)	1.05 (0.99–1.11)
2018/02/13^d^	31.69	33.44	1.76 (−0.14–3.69)	1.06 (1.00–1.12)
2018/02/14	32.64	34.70	2.06 (0.13–4.03)	1.06 (1.00–1.13)
2018/02/15	33.86	35.97	2.11 (0.15–4.11)	1.06 (1.00–1.12)
2018/02/16	34.90	37.39	2.49 (0.49–4.53)	1.07 (1.01–1.13)
2018/02/17	35.95	38.27	2.32 (0.30–4.39)	1.06 (1.01–1.12)
2018/02/18	36.76	39.75	2.99 (0.93–5.09)	1.08 (1.02–1.14)
2018/02/19	38.03	41.59	3.57 (1.46–5.71)	1.09 (1.04–1.15)
2018/02/20	39.14	43.48	4.35 (2.20–6.53)	1.11 (1.06–1.17)

*Abbreviation*: *CI* Confidence interval

^a^ Only 2 weeks before and 1 week after the carnival in 2018 are presented, full presentation of all the available data can be found in Table S4

^b^ Cumulative cases of influenza-related hospitalizations per 100,000 inhabitants

^c^ Compared with non-carnival region

^d^ Carnival period

### Distributions of COVID-19 cases with time between regions in the Netherlands

**Fig. 3:**
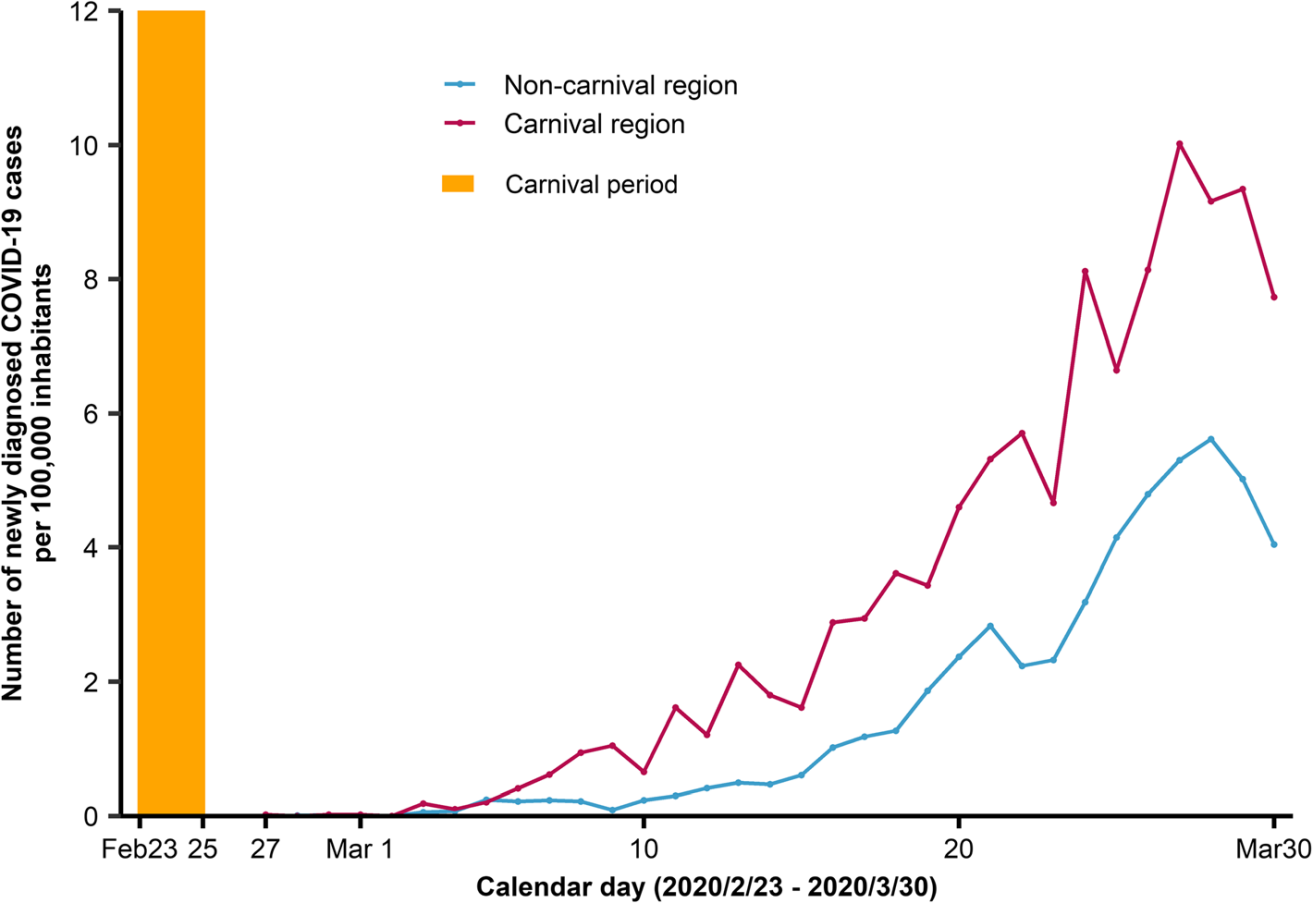
Daily distribution of newly diagnosed COVID-19 cases in the Netherlands

Carnival in 2020 occurred 4 days before the first case of COVID-19 in the Netherlands was reported (from the carnival region). The (virtually zero) increase of new COVID-19 cases kept the same pattern in the first 7 days after the first COVID-19 case was reported, while after that the increase of COVID-19 cases in the carnival region began to exceed that in the non-carnival region (Fig. 3, Figure S2, Table 3, and Table S5).

**Table 3:** Increase of COVID-19 cases per 100,000 inhabitants in the Netherlands

Day^a^	Cumulative Cases^b^		Absolute rate difference^c^ (95% CI)	Rate ratio^c^ (95% CI)
	Non-carnival region	Carnival region		
2020/02/23^d^	–	–	–	–
2020/02/24^d^	–	–	–	–
2020/02/25^d^	–	–	–	–
2020/02/26	–	–	–	–
2020/02/27	0	0.02	0.02 (−0.01–0.11)	13.18 (0.13-Inf)
2020/02/28	0.01	0.02	0.01 (−0.03–0.10)	2.53 (0.05–134.09)
2020/02/29	0.02	0.04	0.02 (−0.04–0.12)	1.74 (0.17–12.81)
2020/03/01	0.03	0.06	0.03 (−0.04–0.15)	1.93 (0.31–10.18)
2020/03/02	0.03	0.06	0.03 (−0.04–0.15)	1.93 (0.31–10.18)
2020/03/03	0.09	0.25	0.16 (0.02–0.34)	2.76 (1.15–6.71)
2020/03/04	0.16	0.35	0.19 (0.02–0.40)	2.16 (1.08–4.26)
2020/03/05	0.40	0.55	0.15 (−0.08–0.42)	1.37 (0.83–2.21)
2020/03/06	0.62	0.96	0.34 (0.04–0.68)	1.55 (1.06–2.24)
2020/03/07	0.86	1.57	0.72 (0.34–1.14)	1.84 (1.36–2.48)
2020/03/08	1.07	2.51	1.44 (0.97–1.96)	2.34 (1.82–3.01)
2020/03/09	1.16	3.56	2.40 (1.85–2.99)	3.06 (2.45–3.84)
2020/03/10	1.40	4.21	2.82 (2.22–3.46)	3.02 (2.46–3.71)
2020/03/11	1.69	5.83	4.13 (3.43–4.88)	3.44 (2.87–4.12)
2020/03/12	2.11	7.03	4.93 (4.15–5.75)	3.34 (2.84–3.93)
2020/03/13	2.60	9.28	6.69 (5.80–7.62)	3.57 (3.09–4.13)
2020/03/14	3.07	11.08	8.02 (7.05–9.03)	3.61 (3.17–4.13)
2020/03/15	3.67	12.70	9.03 (7.98–10.11)	3.46 (3.06–3.91)

*Abbreviation*: *CI* Confidence interval

^a^ Only the first 3 weeks after the first date of the carnival in 2020 are presented in this Table. Full presentation of all the available data can be found in Table S5

^b^ Cumulative cases of COVID-19 cases per 100,000 inhabitants

^c^ Compared with non-carnival region

^d^ Carnival period

## Discussion

In order to assess the association between crowding and transmission patterns of COVID-19 like viral respiratory infectious diseases, we explored the association of carnival (as a proxy for mass gatherings) with the transmission of influenza by comparing the daily increase of new influenza-related hospital admissions before and after carnival between carnival and non-carnival regions with the assumption that influenza transmissibility (an airborne disease) is similar to COVID-19. Influenza-related hospital admissions increased shortly after carnival celebrations which lasted for about 2 weeks but did not occur in the non-carnival regions.

Since data on whether an individual participated in the carnival event was unavailable, we identified regions where carnival was celebrated or not and used this categorization as a proxy of the exposure in the study. As far as we know, this method has not been used previously to study the potential effect of a gathering event. We assumed most individuals who lived in the carnival region would participate in the carnival event, if so the association we observed might be underestimated.

It is not surprising to find that mass gatherings can accelerate the spread of influenza, given that the infection is mainly transmitted via both large and small particles produced by sneezing and coughing [17, 18]. A lesson learned from many outbreaks leading to epidemics of infectious diseases is that forms of isolation or quarantine, such as bans on public gathering, can at least slow an epidemic [19]. As was shown in a study on the 2013 Hajj, crowding conditions can increase the risk of acquisition of pathogens including rhinovirus, coronavirus, and influenza [20]. However, it is uncertain whether such evidence does directly apply to COVID-19, since the scales of gatherings described in these studies may not be similar to that of carnival, which mainly refers to gatherings at community levels, rather than a much larger influx of people across countries. In addition, all these evidences, though intuitive, seems all we currently know about the association between crowding, as the opposite of social distancing, and transmission of viral respiratory infectious diseases. While the public and policymakers need more to make decisions on some important issues, such as when to start/stop the measure of social distancing, and how strict it should be. In our study, no difference in the increase of influenza cases was observed between regions until shortly before carnival. This slight increase of influenza cases before carnival in the carnival region could be just random variation, but could also be related to intermittent pre-carnival festivities before the official carnival arrives (11–13 February in 2018). After the official carnival dates, a drastic increase of new influenza cases was observed in the carnival region, which lasted for 2 weeks.

Our observational study cannot answer why the potential effect of carnival on influenza lasted for about 2 weeks. A speculation, yet to validate, is that the duration of the potential effect of a gathering event is related to the scale and duration of the gathering, the proportion of people at risk of infection, and the contagiousness of the infectious disease itself. Since we focused on a mass gathering at community level of which the participants were mainly the local inhabitants (compared with mass gatherings with a much larger influx of people across countries), the typical characteristics of the infectious disease such as incubation period and the duration of symptoms may dominate the timespan of the effect. The incubation period, namely the time elapsed between exposure to the virus and when symptoms and signs are first apparent, is 1 to 4 days (average 2 days) for influenza [21]. Since influenza is a mild disease for the majority of the public, usually no social distancing measures were taken and patients with symptoms could still spread the pathogen. Therefore, the duration of shedding will also be one of the determinants. Shedding of influenza virus can be detected one-half to 1 day after exposure to the virus, peaks on the second day, and usually ceases within 7 days with an average duration of shedding of about 5 days [22]. In addition, children, the elderly, and patients with chronic illnesses have longer periods of shedding [23, 24]. Also, we studied influenza related hospital admissions instead of the onset of the illness, which occurs later peak than the appearance of symptoms.

Whether what we found in influenza holds true for COVID-19 is more speculative as we know (as compared with influenza) yet little about the contagiousness, incubation period and disease severity of COVID-19 [25]. Nevertheless, from the point of view of virology, SARS-CoV-2 is more similar to SARS-CoV and Middle East respiratory syndrome coronavirus (MERS-CoV) [26, 27] than to influenza virus. Although understanding of the transmission pattern of COVID-19 is still incomplete, it seems to have a longer incubation period than influenza, of 4–14 days [28, 29], and is likely to be more contagious when evaluated by basic reproduction number [30, 31]. The duration of viral shedding is also longer than that of influenza, though the range is quite wide (8 to 37 days) according to different reports [32, 33]. Therefore, if the carnival effect we observed for influenza also exists for COVID-19, the difference should emerge longer after carnival and last longer, which seems consistent with our observations. However, such an observation should be cautiously interpreted because of the absence of COVID-19 cases before the carnival in 2020 and the relatively short follow up time of COVID-19 compared with its incubation period and duration of viral shedding due to the limitation of publicly available COVID-19 data in the Netherlands. The initial investigation on COVID-19 in our study could only provide some potential clues, yet further data are needed to confirm this.

Strengths of our study include that this is a nationwide study and that the categorization of carnival region or non-carnival region was determined at municipal levels and updated numbers of inhabitants between regions were taken into consideration. There are some potential limitations to our study. Frist, influenza was identified based on ICD codes which might cause measurement error. However, the influenza cases we identified in the study were close to those being identified in the annual report of influenza from RIVM [14]. Second, the increase of new influenza cases we observed in the carnival region after carnival might be related to the delay of influenza patients’ visiting hospitals, but since influenza related admissions are usually acute and cannot be delayed, and a continuous increase of new influenza cases could still be observed during the carnival, this concern should not have obfuscated our results. Third, confounding cannot be ruled out in our study. Although overall the two regions shared broadly the same baseline characteristics, slight differences in some characteristics still existed and could have influenced the results (for example, the carnival region had a higher proportion of people aged above 45 years). Due to data limitations, further analysis which could adjust for potential confounders was unavailable. Fourth, regional differences in some systems-level variables, such as test strategy and public health policy, could be a source of confounding. However, for influenza-related hospitalization, test strategy should not be an issue as testing for influenza is universal throughout the Netherlands. For COVID-19, during the study period the test strategy in the Netherlands was mainly based on severe symptomatology. Therefore, we do not expect that testing rate and other health policies influenced regional results on influenza and COVID-19. Fifth, for people who participate in a carnival event, the majority are local inhabitants, but of course it is possible that people (such as tourists) might move in from a non-carnival region to join the event. As both the region of an influenza- and COVID-19 case is based on the region where the patient is registered to live, a person from a non-carnival region could have joined the carnival event and got infected. If so the influenza or COVID-19 case would be counted as from the non-carnival region, but given that only a minority of people outside the local region visit carnival, we consider that this aspect has not materially affected our results. Last, we only investigated the 2017/2018 influenza epidemic in the Netherlands, and it remains unknown whether our conclusion holds true for influenza epidemics in other years. The reasons why we only analyzed the 2017/2018 influenza epidemic were 1) data in that epidemic was the most recent data that we could obtain; 2) the 2017/2018 influenza epidemic in the Netherlands happened to be the worst influenza epidemic of at least the last 5 years (in terms of number of influenza cases and the duration that the epidemic lasted); 3) the carnival event in 2018 occurred near the peak of the influenza epidemic. Without the above conditions, we think it would be difficult to figure out whether the observed results (either with or without difference) between regions was related to the carnival event or other factors (such as a lack of statistical power).

## Conclusions

In this study, a mass gathering event (carnival) was associated with aggravating the spread of viral respiratory infectious diseases.

## Supplementary information

**Additional file 1: Table S1.** Categorization of carnival region and non-carnival region at municipal level in 2018. **Table S2.** Categorization of carnival region and non-carnival region at municipal level in 2019. **Table S3.** Comparison of regional statistics between non-carnival region and carnival region in 2019. **Table S4.** Influenza-related hospitalizations per 100,000 inhabitants in the 2017/2018 influenza epidemic in the Netherlands. **Table S5.** Increase of COVID-19 cases per 100,000 inhabitants in the Netherlands. **Figure S1.** Daily distribution of cumulative cases of influenza-related hospitalizations during the 2017/2018 influenza epidemic in the Netherlands. **Figure S2.** Daily distribution of cumulative cases of COVID-19 in the Netherlands.

## Data Availability

The datasets analyzed during the current study for influenza are not publicly available. Application for access to the data for research purposes would only be approved by Statistics Netherlands (“Centraal Bureau voor de Statistiek”, CBS) when the applicant complies with the criteria for access to the data. Detailed information about applying for access to the data can be found at https://www.cbs.nl/en-gb/our-services/customised-services-microdata/microdata-conducting-your-own-research/applying-for-access-to-microdata. Data on COVID-19 diagnoses can be accessed from CoronaWatchNL12 (https://github.com/J535D165/CoronaWatchNL).

## Abbreviations

COVID-19: Coronavirus disease 2019
SARS-CoV-2: Severe acute respiratory syndrome coronavirus 2
SARS: Severe acute respiratory syndrome
CBS: Centraal Bureau voor de Statistiek
RIVM: Dutch National Institute for Public Health and the Environment
ICD: International Classification of Diseases
MERS-CoV: Middle East respiratory syndrome coronavirus
CI: Confidence interval
