# Supplementary material for: Why crowding matters in the time of COVID-19 pandemic? - a lesson from the carnival effect on the 2017/2018 influenza epidemic in the Netherlands
Source: BMC Public Health. 2020 Oct 6;20:1516. doi: 10.1186/s12889-020-09612-6 (PMC7537972; doi:10.1186/s12889-020-09612-6)

**Supplemental materials**

**Supplemental Tables**

**Table S1. Categorization of carnival region and non-carnival region at municipal level in 2018**

| Type of region | Municipalities |
| --- | --- |
| Carnival region | Almelo; Borne; Enschede; Haaksbergen; Hellendoorn; Hengelo; Losser; Oldenzaal; Tubbergen; Wierden; Arnhem; Duiven; Nijmegen; Westervoort; Zevenaar; Hulst; Aalburg; Asten; Baarle-Nassau; Bergen op Zoom; Best; Boekel; Boxmeer; Boxtel; Breda; Deurne; Dongen; Eersel; Eindhoven; Etten-Leur; Geertruidenberg; Gilze en Rijen; Goirle; Grave; Haaren; Helmond; 's-Hertogenbosch; Heusden; Hilvarenbeek; Loon op Zand; Mill en Sint Hubert; Nuenen, Gerwen en Nederwetten; Oirschot; Oisterwijk; Oosterhout; Oss; Rucphen; Sint-Michielsgestel; Someren; Son en Breugel; Steenbergen; Tilburg; Uden; Valkenswaard; Veldhoven; Vught; Waalre; Waalwijk; Werkendam; Woensdrecht; Woudrichem; Zundert; Onderbanken; Landgraaf; Beek; Beesel; Bergen (L.); Brunssum; Gennep; Heerlen; Kerkrade; Maastricht; Meerssen; Mook en Middelaar; Nederweert; Nuth; Roermond; Schinnen; Simpelveld; Stein; Vaals; Venlo; Venray; Voerendaal; Weert; Valkenburg aan de Geul; Horst aan de Maas; Leudal; Maasgouw; Gemert-Bakel; Halderberge; Heeze-Leende; Laarbeek; Reusel-De Mierden; Roerdalen; Roosendaal; Cuijk; Landerd; Twenterand; Sint Anthonis; Lingewaard; Cranendonck; Moerdijk; Echt-Susteren; Sluis; Drimmelen; Bernheze; Alphen-Chaam; Bergeijk; Bladel; Gulpen-Wittem; Overbetuwe; Hof van Twente; Rijssen-Holten; Geldrop-Mierlo; Dinkelland; Sittard-Geleen; Peel en Maas; Eijsden-Margraten; Meierijstad; Montferland |
| Non-carnival region | Appingedam; Bedum; Ten Boer; Delfzijl; Groningen; Grootegast; Haren; Leek; Loppersum; Marum; Almere; Stadskanaal; Veendam; Zeewolde; Winsum; Zuidhorn; Dongeradeel; Achtkarspelen; Ameland; Harlingen; Heerenveen; Kollumerland en Nieuwkruisland; Leeuwarden; Ooststellingwerf; Opsterland; Schiermonnikoog; Smallingerland; Terschelling; Vlieland; Weststellingwerf; Assen; Coevorden; Emmen; Hoogeveen; Meppel; Dalfsen; Deventer; Hardenberg; Kampen; Noordoostpolder; Ommen; Raalte; Staphorst; Urk; Zwolle; Aalten; Apeldoorn; Barneveld; Beuningen; Brummen; Buren; Culemborg; Doesburg; Doetinchem; Druten; Ede; Elburg; Epe; Ermelo; Geldermalsen; Harderwijk; Hattem; Heerde; Heumen; Lochem; Maasdriel; Nijkerk; Oldebroek; Putten; Renkum; Rheden; Rozendaal; Scherpenzeel; Tiel; Voorst; Wageningen; Winterswijk; Wijchen; Zaltbommel; Zutphen; Nunspeet; Dronten; Neerijnen; Amersfoort; Baarn; De Bilt; Bunnik; Bunschoten; Eemnes; Houten; Leusden; Lopik; Montfoort; Renswoude; Rhenen; Soest; Utrecht; Veenendaal; Woudenberg; Wijk bij Duurstede; IJsselstein; Zeist; Nieuwegein; Aalsmeer; Alkmaar; Amstelveen; Amsterdam; Beemster; Bergen (NH.); Beverwijk; Blaricum; Bloemendaal; Castricum; Diemen; Edam-Volendam; Enkhuizen; Haarlem; Haarlemmerliede en Spaarnwoude; Haarlemmermeer; Heemskerk; Heemstede; Heerhugowaard; Heiloo; Den Helder; Hilversum; Hoorn; Huizen; Landsmeer; Langedijk; Laren; Medemblik; Oostzaan; Opmeer; Ouder-Amstel; Purmerend; Schagen; Texel; Uitgeest; Uithoorn; Velsen; Weesp; Zandvoort; Zaanstad; Alblasserdam; Alphen aan den Rijn; Barendrecht; Drechterland; Brielle; Capelle aan den IJssel; Delft; Dordrecht; Gorinchem; Gouda; s-Gravenhage; Hardinxveld-Giessendam; Hellevoetsluis; Hendrik-Ido-Ambacht; Stede Broec; Hillegom; Katwijk; Krimpen aan den IJssel; Leerdam; Leiden; Leiderdorp; Lisse; Maassluis; Nieuwkoop; Noordwijk; Noordwijkerhout; Oegstgeest; Oud-Beijerland; Binnenmaas; Korendijk; Oudewater; Papendrecht; Ridderkerk; Rotterdam; Rijswijk; Schiedam; Sliedrecht; Cromstrijen; Albrandswaard; Westvoorne; Strijen; Vianen; Vlaardingen; Voorschoten; Waddinxveen; Wassenaar; Woerden; Zoetermeer; Zoeterwoude; Zwijndrecht; Borsele; Goes; West Maas en Waal; Kapelle; Middelburg; Giessenlanden; Reimerswaal; Zederik; Terneuzen; Tholen; Veere; Vlissingen; Lingewaal; De Ronde Venen; Tytsjerksteradiel; Pekela; Waterland; Wormerland; Lelystad; Oude IJsselstreek; Teylingen; Utrechtse Heuvelrug; Oost Gelre; Koggenland; Lansingerland; Eemsmond; De Marne; Schouwen-Duiveland; Aa en Hunze; Borger-Odoorn; De Wolden; Noord-Beveland; Wijdemeren; Noordenveld; Westerveld; Steenwijkerland; Ferwerderadiel; Tynaarlo; Midden-Drenthe; Neder-Betuwe; Olst-Wijhe; Westland; Midden-Delfland; Berkelland; Bronckhorst; Kaag en Braassem; Dantumadiel; Zuidplas; Oldambt; Zwartewaterland; Súdwest-Fryslân; Bodegraven-Reeuwijk; Stichtse Vecht; Hollands Kroon; Leidschendam-Voorburg; Goeree-Overflakkee; Pijnacker-Nootdorp; Molenwaard; Nissewaard; Krimpenerwaard; De Fryske Marren; Gooise Meren; Berg en Dal; Waadhoeke; Westerwolde; Midden-Groningen |

**Table S2. Categorization of carnival region and non-carnival region at municipal level in 2019**

| Type of region | Municipalities |
| --- | --- |
| Carnival region | Almelo; Borne; Enschede; Haaksbergen; Hellendoorn; Hengelo; Losser; Oldenzaal; Tubbergen; Wierden; Arnhem; Duiven; Nijmegen; Westervoort; Zevenaar; Hulst; Asten; Baarle-Nassau; Bergen op Zoom; Best; Boekel; Boxmeer; Boxtel; Breda; Deurne; Dongen; Eersel; Eindhoven; Etten-Leur; Geertruidenberg; Gilze en Rijen; Goirle; Grave; Haaren; Helmond; 's-Hertogenbosch; Heusden; Hilvarenbeek; Loon op Zand; Mill en Sint Hubert; Nuenen, Gerwen en Nederwetten; Oirschot; Oisterwijk; Oosterhout; Oss; Rucphen; Sint-Michielsgestel; Someren; Son en Breugel; Steenbergen; Tilburg; Uden; Valkenswaard; Veldhoven; Vught; Waalre; Waalwijk; Woensdrecht; Zundert; Landgraaf; Beek; Beesel; Bergen (L.); Brunssum; Gennep; Heerlen; Kerkrade; Maastricht; Meerssen; Mook en Middelaar; Nederweert; Roermond; Simpelveld; Stein; Vaals; Venlo; Venray; Voerendaal; Weert; Valkenburg aan de Geul; Horst aan de Maas; Leudal; Maasgouw; Gemert-Bakel; Halderberge; Heeze-Leende; Laarbeek; Reusel-De Mierden; Roerdalen; Roosendaal; Cuijk; Landerd; Twenterand; Sint Anthonis; Lingewaard; Cranendonck; Moerdijk; Echt-Susteren; Sluis; Drimmelen; Bernheze; Alphen-Chaam; Bergeijk; Bladel; Gulpen-Wittem; Overbetuwe; Hof van Twente; Rijssen-Holten; Geldrop-Mierlo; Dinkelland; Sittard-Geleen; Peel en Maas; Eijsden-Margraten; Meierijstad; Beekdaelen; Montferland; Altena |
| Non-carnival region | Appingedam; Delfzijl; Groningen; Loppersum; Almere; Stadskanaal; Veendam; Zeewolde; Achtkarspelen; Ameland; Harlingen; Heerenveen; Leeuwarden; Ooststellingwerf; Opsterland; Schiermonnikoog; Smallingerland; Terschelling; Vlieland; Weststellingwerf; Assen; Coevorden; Emmen; Hoogeveen; Meppel; Dalfsen; Deventer; Hardenberg; Kampen; Noordoostpolder; Ommen; Raalte; Staphorst; Urk; Zwolle; Aalten; Apeldoorn; Barneveld; Beuningen; Brummen; Buren; Culemborg; Doesburg; Doetinchem; Druten; Ede; Elburg; Epe; Ermelo; Harderwijk; Hattem; Heerde; Heumen; Lochem; Maasdriel; Nijkerk; Oldebroek; Putten; Renkum; Rheden; Rozendaal; Scherpenzeel; Tiel; Voorst; Wageningen; Winterswijk; Wijchen; Zaltbommel; Zutphen; Nunspeet; Dronten; Amersfoort; Baarn; De Bilt; Bunnik; Bunschoten; Eemnes; Houten; Leusden; Lopik; Montfoort; Renswoude; Rhenen; Soest; Utrecht; Veenendaal; Woudenberg; Wijk bij Duurstede; IJsselstein; Zeist; Nieuwegein; Aalsmeer; Alkmaar; Amstelveen; Amsterdam; Beemster; Bergen (NH.); Beverwijk; Blaricum; Bloemendaal; Castricum; Diemen; Edam-Volendam; Enkhuizen; Haarlem; Haarlemmermeer; Heemskerk; Heemstede; Heerhugowaard; Heiloo; Den Helder; Hilversum; Hoorn; Huizen; Landsmeer; Langedijk; Laren; Medemblik; Oostzaan; Opmeer; Ouder-Amstel; Purmerend; Schagen; Texel; Uitgeest; Uithoorn; Velsen; Weesp; Zandvoort; Zaanstad; Alblasserdam; Alphen aan den Rijn; Barendrecht; Drechterland; Brielle; Capelle aan den IJssel; Delft; Dordrecht; Gorinchem; Gouda; s-Gravenhage; Hardinxveld-Giessendam; Hellevoetsluis; Hendrik-Ido-Ambacht; Stede Broec; Hillegom; Katwijk; Krimpen aan den IJssel; Leiden; Leiderdorp; Lisse; Maassluis; Nieuwkoop; Noordwijk; Oegstgeest; Oudewater; Papendrecht; Ridderkerk; Rotterdam; Rijswijk; Schiedam; Sliedrecht; Albrandswaard; Westvoorne; Vlaardingen; Voorschoten; Waddinxveen; Wassenaar; Woerden; Zoetermeer; Zoeterwoude; Zwijndrecht; Borsele; Goes; West Maas en Waal; Kapelle; Middelburg; Reimerswaal; Terneuzen; Tholen; Veere; Vlissingen; De Ronde Venen; Tytsjerksteradiel; Pekela; Waterland; Wormerland; Lelystad; Oude IJsselstreek; Teylingen; Utrechtse Heuvelrug; Oost Gelre; Koggenland; Lansingerland; Schouwen-Duiveland; Aa en Hunze; Borger-Odoorn; De Wolden; Noord-Beveland; Wijdemeren; Noordenveld; Westerveld; Steenwijkerland; Tynaarlo; Midden-Drenthe; Neder-Betuwe; Olst-Wijhe; Westland; Midden-Delfland; Berkelland; Bronckhorst; Kaag en Braassem; Dantumadiel; Zuidplas; Oldambt; Zwartewaterland; Súdwest-Fryslân; Bodegraven-Reeuwijk; Stichtse Vecht; Hollands Kroon; Leidschendam-Voorburg; Goeree-Overflakkee; Pijnacker-Nootdorp; Nissewaard; Krimpenerwaard; De Fryske Marren; Gooise Meren; Berg en Dal; Waadhoeke; Westerwolde; Midden-Groningen; West Betuwe; Vijfheerenlanden; Hoeksche Waard; Het Hogeland; Westerkwartier; Noardeast-Fryslân; Molenlanden |

**Table S3. Comparison of regional statistics between non-carnival region and carnival region in 2019**

| Variables | Non-carnival region | Carnival region |
| --- | --- | --- |
| No. of inhabitants | 12391220 | 4890943 |
| No. inhabitants per km^2†^ | 496 | 439 |
| Degree of urbanity^†‡^ | 3 | 4 |
| No. mortality | - | - |
| Sex |  |  |
| Male | 6137974 (49.5) | 2443112 (50.0) |
| Female | 6253246 (50.5) | 2447831 (50.0) |
| Age groups (year) |  |  |
| 0-15 | 2016663 (16.3) | 723156 (14.8) |
| 15-25 | 1529135 (12.3) | 602809 (12.3) |
| 25-45 | 3107821 (25.1) | 1147629 (23.5) |
| 45-65 | 3430117 (27.7) | 1410829 (28.8) |
| ≥65 | 2307484 (18.6) | 1006520 (20.6) |
| Marital status |  |  |
| Unmarried | 6103527 (49.3) | 2270606 (46.4) |
| Married | 4733308 (38.2) | 1973246 (40.3) |
| Separated | 964076 (7.8) | 378957 (7.7) |
| Widowed | 590309 (4.8) | 268134 (5.5) |
| Type of family home^†^ |  |  |
| Single family home (%) | 80 | 85 |
| Multiple family home (%) | 20 | 15 |

† Median of the region

‡ According to the environmental address density, an urban class has been assigned to every neighborhood, district or municipality. The following class division has been used: 1, very strong urban> = 2 500 addresses per km²; 2, strongly urban 1 500 - 2 500 addresses per km²; 3, moderately urban 1 000 - 1 500 addresses per km²; 4, few urban 500 - 1 000 addresses per km²; 5, non-urban <500 addresses per km².

**Table S4. Influenza-related hospitalizations per 100,000 inhabitants in the 2017/2018 influenza epidemic in the Netherlands**

| Day | Cumulative Cases^†^ | | Absolute rate difference^Ω^  (95% CI) | Rate ratio^Ω^  (95% CI) |
| --- | --- | --- | --- | --- |
|  | Non-carnival region | Carnival region |  |  |
| 2017/10/2 | 0.01 | 0.02 | 0.01 (-0.03-0.11) | 2.53 (0.05-133.76) |
| 2017/10/3 | 0.02 | 0.02 | 0.01 (-0.04-0.1) | 1.37 (0.03-19.08) |
| 2017/10/4 | 0.02 | 0.08 | 0.06 (-0.02-0.19) | 3.32 (0.63-20.58) |
| 2017/10/5 | 0.02 | 0.1 | 0.08 (-0.01-0.22) | 4.12 (0.89-24.54) |
| 2017/10/6 | 0.02 | 0.1 | 0.08 (-0.01-0.22) | 4.12 (0.89-24.54) |
| 2017/10/7 | 0.03 | 0.1 | 0.07 (-0.02-0.21) | 3.13 (0.73-14.65) |
| 2017/10/8 | 0.04 | 0.1 | 0.06 (-0.03-0.2) | 2.53 (0.62-10.23) |
| 2017/10/9 | 0.05 | 0.1 | 0.06 (-0.04-0.19) | 2.12 (0.54-7.79) |
| 2017/10/10 | 0.06 | 0.1 | 0.05 (-0.05-0.19) | 1.82 (0.48-6.26) |
| 2017/10/11 | 0.06 | 0.1 | 0.04 (-0.06-0.18) | 1.6 (0.43-5.22) |
| 2017/10/12 | 0.07 | 0.12 | 0.05 (-0.05-0.2) | 1.7 (0.52-5.09) |
| 2017/10/13 | 0.08 | 0.12 | 0.04 (-0.06-0.19) | 1.53 (0.48-4.44) |
| 2017/10/14 | 0.08 | 0.12 | 0.04 (-0.06-0.19) | 1.53 (0.48-4.44) |
| 2017/10/15 | 0.08 | 0.14 | 0.06 (-0.05-0.22) | 1.78 (0.6-4.97) |
| 2017/10/16 | 0.09 | 0.16 | 0.08 (-0.04-0.24) | 1.85 (0.67-4.86) |
| 2017/10/17 | 0.09 | 0.16 | 0.08 (-0.04-0.24) | 1.85 (0.67-4.86) |
| 2017/10/18 | 0.1 | 0.18 | 0.09 (-0.04-0.26) | 1.9 (0.73-4.76) |
| 2017/10/19 | 0.1 | 0.21 | 0.11 (-0.02-0.29) | 2.11 (0.84-5.17) |
| 2017/10/20 | 0.11 | 0.21 | 0.09 (-0.04-0.27) | 1.81 (0.74-4.26) |
| 2017/10/21 | 0.11 | 0.21 | 0.09 (-0.04-0.27) | 1.81 (0.74-4.26) |
| 2017/10/22 | 0.14 | 0.23 | 0.09 (-0.05-0.27) | 1.64 (0.71-3.63) |
| 2017/10/23 | 0.17 | 0.23 | 0.06 (-0.09-0.24) | 1.33 (0.59-2.82) |
| 2017/10/24 | 0.19 | 0.23 | 0.04 (-0.11-0.23) | 1.22 (0.54-2.54) |
| 2017/10/25 | 0.19 | 0.23 | 0.04 (-0.11-0.23) | 1.22 (0.54-2.54) |
| 2017/10/26 | 0.2 | 0.23 | 0.02 (-0.13-0.21) | 1.12 (0.5-2.31) |
| 2017/10/27 | 0.21 | 0.25 | 0.04 (-0.12-0.23) | 1.18 (0.55-2.36) |
| 2017/10/28 | 0.23 | 0.25 | 0.02 (-0.14-0.22) | 1.09 (0.51-2.17) |
| 2017/10/29 | 0.25 | 0.27 | 0.02 (-0.15-0.22) | 1.07 (0.52-2.06) |
| 2017/10/30 | 0.26 | 0.29 | 0.03 (-0.14-0.24) | 1.11 (0.55-2.11) |
| 2017/10/31 | 0.28 | 0.31 | 0.03 (-0.14-0.25) | 1.12 (0.57-2.08) |
| 2017/11/1 | 0.28 | 0.33 | 0.05 (-0.13-0.27) | 1.2 (0.62-2.19) |
| 2017/11/2 | 0.28 | 0.33 | 0.05 (-0.13-0.27) | 1.16 (0.61-2.12) |
| 2017/11/3 | 0.29 | 0.33 | 0.04 (-0.14-0.26) | 1.13 (0.59-2.06) |
| 2017/11/4 | 0.29 | 0.33 | 0.04 (-0.14-0.26) | 1.13 (0.59-2.06) |
| 2017/11/5 | 0.31 | 0.37 | 0.06 (-0.13-0.29) | 1.2 (0.65-2.13) |
| 2017/11/6 | 0.32 | 0.37 | 0.05 (-0.14-0.29) | 1.17 (0.64-2.07) |
| 2017/11/7 | 0.32 | 0.37 | 0.05 (-0.14-0.29) | 1.17 (0.64-2.07) |
| 2017/11/8 | 0.32 | 0.37 | 0.05 (-0.15-0.28) | 1.14 (0.62-2.01) |
| 2017/11/9 | 0.33 | 0.37 | 0.04 (-0.16-0.27) | 1.12 (0.61-1.96) |
| 2017/11/10 | 0.35 | 0.39 | 0.04 (-0.16-0.28) | 1.12 (0.62-1.94) |
| 2017/11/11 | 0.35 | 0.41 | 0.06 (-0.14-0.3) | 1.18 (0.66-2.03) |
| 2017/11/12 | 0.35 | 0.41 | 0.06 (-0.14-0.3) | 1.18 (0.66-2.03) |
| 2017/11/13 | 0.36 | 0.41 | 0.06 (-0.15-0.3) | 1.15 (0.65-1.97) |
| 2017/11/14 | 0.37 | 0.43 | 0.06 (-0.15-0.31) | 1.16 (0.66-1.96) |
| 2017/11/15 | 0.39 | 0.43 | 0.04 (-0.17-0.29) | 1.11 (0.64-1.87) |
| 2017/11/16 | 0.41 | 0.43 | 0.03 (-0.18-0.28) | 1.07 (0.61-1.79) |
| 2017/11/17 | 0.42 | 0.43 | 0.01 (-0.2-0.26) | 1.03 (0.59-1.71) |
| 2017/11/18 | 0.44 | 0.43 | -0.01 (-0.22-0.24) | 0.99 (0.57-1.64) |
| 2017/11/19 | 0.45 | 0.45 | 0.01 (-0.21-0.26) | 1.02 (0.59-1.67) |
| 2017/11/20 | 0.48 | 0.47 | 0 (-0.23-0.26) | 0.99 (0.59-1.61) |
| 2017/11/21 | 0.5 | 0.51 | 0.02 (-0.21-0.29) | 1.04 (0.63-1.67) |
| 2017/11/22 | 0.5 | 0.55 | 0.05 (-0.19-0.33) | 1.1 (0.68-1.75) |
| 2017/11/23 | 0.53 | 0.57 | 0.05 (-0.19-0.33) | 1.09 (0.68-1.71) |
| 2017/11/24 | 0.53 | 0.6 | 0.07 (-0.18-0.35) | 1.13 (0.71-1.76) |
| 2017/11/25 | 0.54 | 0.62 | 0.07 (-0.18-0.36) | 1.14 (0.72-1.76) |
| 2017/11/26 | 0.56 | 0.64 | 0.08 (-0.18-0.37) | 1.14 (0.72-1.75) |
| 2017/11/27 | 0.58 | 0.64 | 0.05 (-0.2-0.35) | 1.09 (0.7-1.67) |
| 2017/11/28 | 0.58 | 0.68 | 0.09 (-0.17-0.39) | 1.16 (0.75-1.76) |
| 2017/11/29 | 0.59 | 0.68 | 0.09 (-0.18-0.39) | 1.15 (0.74-1.74) |
| 2017/11/30 | 0.63 | 0.68 | 0.05 (-0.21-0.36) | 1.09 (0.7-1.64) |
| 2017/12/1 | 0.68 | 0.7 | 0.02 (-0.25-0.33) | 1.03 (0.67-1.53) |
| 2017/12/2 | 0.71 | 0.72 | 0.01 (-0.26-0.33) | 1.02 (0.67-1.52) |
| 2017/12/3 | 0.74 | 0.74 | 0 (-0.28-0.32) | 1 (0.66-1.48) |
| 2017/12/4 | 0.76 | 0.74 | -0.02 (-0.3-0.29) | 0.97 (0.64-1.43) |
| 2017/12/5 | 0.8 | 0.78 | -0.01 (-0.3-0.31) | 0.98 (0.66-1.43) |
| 2017/12/6 | 0.84 | 0.8 | -0.03 (-0.33-0.3) | 0.96 (0.65-1.39) |
| 2017/12/7 | 0.88 | 0.84 | -0.03 (-0.33-0.3) | 0.96 (0.66-1.38) |
| 2017/12/8 | 0.94 | 0.86 | -0.08 (-0.39-0.26) | 0.92 (0.63-1.31) |
| 2017/12/9 | 0.97 | 0.9 | -0.07 (-0.38-0.28) | 0.93 (0.64-1.32) |
| 2017/12/10 | 1 | 0.9 | -0.09 (-0.41-0.26) | 0.91 (0.63-1.28) |
| 2017/12/11 | 1.08 | 0.99 | -0.09 (-0.42-0.27) | 0.91 (0.64-1.27) |
| 2017/12/12 | 1.15 | 1.03 | -0.12 (-0.45-0.25) | 0.9 (0.64-1.24) |
| 2017/12/13 | 1.21 | 1.09 | -0.12 (-0.47-0.26) | 0.9 (0.65-1.23) |
| 2017/12/14 | 1.28 | 1.13 | -0.14 (-0.5-0.24) | 0.89 (0.64-1.21) |
| 2017/12/15 | 1.32 | 1.19 | -0.12 (-0.49-0.27) | 0.91 (0.66-1.23) |
| 2017/12/16 | 1.36 | 1.23 | -0.12 (-0.49-0.28) | 0.91 (0.67-1.22) |
| 2017/12/17 | 1.38 | 1.25 | -0.13 (-0.5-0.28) | 0.91 (0.67-1.22) |
| 2017/12/18 | 1.51 | 1.38 | -0.13 (-0.52-0.29) | 0.91 (0.68-1.21) |
| 2017/12/19 | 1.61 | 1.46 | -0.15 (-0.55-0.29) | 0.91 (0.68-1.19) |
| 2017/12/20 | 1.74 | 1.6 | -0.13 (-0.55-0.32) | 0.92 (0.7-1.2) |
| 2017/12/21 | 1.81 | 1.66 | -0.15 (-0.57-0.32) | 0.92 (0.7-1.19) |
| 2017/12/22 | 1.99 | 1.77 | -0.22 (-0.67-0.26) | 0.89 (0.69-1.14) |
| 2017/12/23 | 2.15 | 1.87 | -0.28 (-0.74-0.21) | 0.87 (0.68-1.1) |
| 2017/12/24 | 2.34 | 2.05 | -0.28 (-0.76-0.23) | 0.88 (0.69-1.1) |
| 2017/12/25 | 2.46 | 2.16 | -0.3 (-0.8-0.22) | 0.88 (0.7-1.1) |
| 2017/12/26 | 2.65 | 2.46 | -0.18 (-0.7-0.37) | 0.93 (0.75-1.15) |
| 2017/12/27 | 2.97 | 2.77 | -0.2 (-0.75-0.39) | 0.93 (0.76-1.14) |
| 2017/12/28 | 3.17 | 3.08 | -0.09 (-0.67-0.53) | 0.97 (0.8-1.18) |
| 2017/12/29 | 3.45 | 3.31 | -0.14 (-0.75-0.49) | 0.96 (0.79-1.15) |
| 2017/12/30 | 3.61 | 3.57 | -0.04 (-0.66-0.62) | 0.99 (0.83-1.18) |
| 2017/12/31 | 3.92 | 3.84 | -0.08 (-0.73-0.6) | 0.98 (0.82-1.16) |
| 2018/1/1 | 4.31 | 4.31 | 0.01 (-0.68-0.73) | 1 (0.85-1.18) |
| 2018/1/2 | 4.8 | 4.64 | -0.16 (-0.87-0.59) | 0.97 (0.83-1.13) |
| 2018/1/3 | 5.27 | 4.85 | -0.42 (-1.16-0.34) | 0.92 (0.79-1.07) |
| 2018/1/4 | 5.64 | 5.3 | -0.34 (-1.1-0.46) | 0.94 (0.81-1.08) |
| 2018/1/5 | 6.1 | 5.77 | -0.33 (-1.13-0.5) | 0.95 (0.82-1.09) |
| 2018/1/6 | 6.42 | 6.1 | -0.32 (-1.14-0.54) | 0.95 (0.83-1.09) |
| 2018/1/7 | 6.73 | 6.49 | -0.24 (-1.09-0.63) | 0.96 (0.84-1.1) |
| 2018/1/8 | 7.07 | 7.02 | -0.04 (-0.92-0.86) | 0.99 (0.87-1.13) |
| 2018/1/9 | 7.41 | 7.39 | -0.02 (-0.91-0.92) | 1 (0.88-1.13) |
| 2018/1/10 | 7.77 | 7.64 | -0.13 (-1.04-0.82) | 0.98 (0.87-1.11) |
| 2018/1/11 | 8.18 | 8.01 | -0.17 (-1.11-0.8) | 0.98 (0.87-1.1) |
| 2018/1/12 | 8.68 | 8.44 | -0.24 (-1.2-0.76) | 0.97 (0.87-1.09) |
| 2018/1/13 | 9.02 | 8.83 | -0.19 (-1.17-0.83) | 0.98 (0.87-1.1) |
| 2018/1/14 | 9.39 | 9.18 | -0.21 (-1.21-0.83) | 0.98 (0.87-1.09) |
| 2018/1/15 | 9.91 | 9.67 | -0.24 (-1.27-0.83) | 0.98 (0.88-1.09) |
| 2018/1/16 | 10.38 | 10.27 | -0.11 (-1.17-0.98) | 0.99 (0.89-1.1) |
| 2018/1/17 | 10.93 | 10.84 | -0.09 (-1.18-1.03) | 0.99 (0.9-1.1) |
| 2018/1/18 | 11.44 | 11.39 | -0.04 (-1.16-1.11) | 1 (0.9-1.1) |
| 2018/1/19 | 12.12 | 12.13 | 0.02 (-1.13-1.2) | 1 (0.91-1.1) |
| 2018/1/20 | 12.57 | 12.46 | -0.11 (-1.28-1.09) | 0.99 (0.9-1.09) |
| 2018/1/21 | 13.1 | 12.89 | -0.21 (-1.4-1.01) | 0.98 (0.9-1.08) |
| 2018/1/22 | 13.84 | 13.82 | -0.02 (-1.25-1.24) | 1 (0.91-1.09) |
| 2018/1/23 | 14.64 | 14.49 | -0.14 (-1.4-1.15) | 0.99 (0.91-1.08) |
| 2018/1/24 | 15.28 | 15.03 | -0.25 (-1.53-1.07) | 0.98 (0.9-1.07) |
| 2018/1/25 | 15.99 | 16.01 | 0.02 (-1.3-1.38) | 1 (0.92-1.09) |
| 2018/1/26 | 16.65 | 17.1 | 0.45 (-0.91-1.85) | 1.03 (0.95-1.11) |
| 2018/1/27 | 17.22 | 17.94 | 0.72 (-0.67-2.15) | 1.04 (0.96-1.13) |
| 2018/1/28 | 17.94 | 18.54 | 0.6 (-0.81-2.06) | 1.03 (0.96-1.12) |
| 2018/1/29 | 18.79 | 19.65 | 0.86 (-0.6-2.35) | 1.05 (0.97-1.13) |
| 2018/1/30 | 19.55 | 20.51 | 0.96 (-0.53-2.48) | 1.05 (0.97-1.13) |
| 2018/1/31 | 20.49 | 21.43 | 0.95 (-0.57-2.5) | 1.05 (0.97-1.12) |
| 2018/2/1 | 21.21 | 22.32 | 1.11 (-0.44-2.69) | 1.05 (0.98-1.13) |
| 2018/2/2 | 22.31 | 23.32 | 1.01 (-0.58-2.63) | 1.05 (0.97-1.12) |
| 2018/2/3 | 22.92 | 24.06 | 1.14 (-0.47-2.78) | 1.05 (0.98-1.12) |
| 2018/2/4 | 23.49 | 24.66 | 1.17 (-0.46-2.83) | 1.05 (0.98-1.12) |
| 2018/2/5 | 24.46 | 25.91 | 1.45 (-0.22-3.16) | 1.06 (0.99-1.13) |
| 2018/2/6 | 25.18 | 26.83 | 1.65 (-0.04-3.38) | 1.07 (1-1.14) |
| 2018/2/7 | 26.03 | 27.59 | 1.57 (-0.15-3.32) | 1.06 (0.99-1.13) |
| 2018/2/8 | 26.9 | 28.64 | 1.74 (-0.02-3.53) | 1.06 (1-1.13) |
| 2018/2/9 | 27.96 | 29.63 | 1.67 (-0.12-3.49) | 1.06 (1-1.13) |
| 2018/2/10 | 28.65 | 30.49 | 1.84 (0.03-3.68) | 1.06 (1-1.13) |
| 2018/2/11 | 29.48 | 31.31 | 1.83 (0-3.7) | 1.06 (1-1.13) |
| 2018/2/12 | 30.69 | 32.25 | 1.57 (-0.3-3.46) | 1.05 (0.99-1.11) |
| 2018/2/13 | 31.69 | 33.44 | 1.76 (-0.14-3.69) | 1.06 (1-1.12) |
| 2018/2/14 | 32.64 | 34.7 | 2.06 (0.13-4.03) | 1.06 (1-1.13) |
| 2018/2/15 | 33.86 | 35.97 | 2.11 (0.15-4.11) | 1.06 (1-1.12) |
| 2018/2/16 | 34.9 | 37.39 | 2.49 (0.49-4.53) | 1.07 (1.01-1.13) |
| 2018/2/17 | 35.95 | 38.27 | 2.32 (0.3-4.39) | 1.06 (1.01-1.12) |
| 2018/2/18 | 36.76 | 39.75 | 2.99 (0.93-5.09) | 1.08 (1.02-1.14) |
| 2018/2/19 | 38.03 | 41.59 | 3.57 (1.46-5.71) | 1.09 (1.04-1.15) |
| 2018/2/20 | 39.14 | 43.48 | 4.35 (2.2-6.53) | 1.11 (1.06-1.17) |
| 2018/2/21 | 40.3 | 45.17 | 4.87 (2.68-7.09) | 1.12 (1.07-1.18) |
| 2018/2/22 | 41.58 | 46.97 | 5.39 (3.16-7.66) | 1.13 (1.07-1.19) |
| 2018/2/23 | 43.02 | 48.68 | 5.66 (3.39-7.96) | 1.13 (1.08-1.19) |
| 2018/2/24 | 43.99 | 50.07 | 6.09 (3.79-8.42) | 1.14 (1.08-1.19) |
| 2018/2/25 | 45 | 51.63 | 6.63 (4.3-9) | 1.15 (1.09-1.2) |
| 2018/2/26 | 46.33 | 53.56 | 7.24 (4.86-9.65) | 1.16 (1.1-1.21) |
| 2018/2/27 | 47.46 | 55.27 | 7.81 (5.4-10.25) | 1.16 (1.11-1.22) |
| 2018/2/28 | 48.57 | 57.26 | 8.69 (6.24-11.18) | 1.18 (1.13-1.23) |
| 2018/3/1 | 49.89 | 58.88 | 9 (6.51-11.52) | 1.18 (1.13-1.23) |
| 2018/3/2 | 51.1 | 60.4 | 9.31 (6.79-11.86) | 1.18 (1.13-1.24) |
| 2018/3/3 | 52.27 | 61.9 | 9.64 (7.09-12.22) | 1.18 (1.13-1.24) |
| 2018/3/4 | 53.53 | 63.4 | 9.87 (7.29-12.48) | 1.18 (1.13-1.24) |
| 2018/3/5 | 54.96 | 65.22 | 10.26 (7.65-12.91) | 1.19 (1.14-1.24) |
| 2018/3/6 | 56.29 | 67.13 | 10.84 (8.19-13.53) | 1.19 (1.14-1.24) |
| 2018/3/7 | 57.61 | 68.8 | 11.19 (8.51-13.91) | 1.19 (1.15-1.24) |
| 2018/3/8 | 58.8 | 70.19 | 11.4 (8.69-14.14) | 1.19 (1.15-1.24) |
| 2018/3/9 | 60.1 | 71.65 | 11.55 (8.81-14.32) | 1.19 (1.14-1.24) |
| 2018/3/10 | 61.06 | 72.66 | 11.6 (8.84-14.39) | 1.19 (1.14-1.24) |
| 2018/3/11 | 61.97 | 73.7 | 11.73 (8.96-14.55) | 1.19 (1.14-1.24) |
| 2018/3/12 | 63.1 | 74.94 | 11.84 (9.04-14.67) | 1.19 (1.14-1.24) |
| 2018/3/13 | 63.93 | 75.74 | 11.81 (8.99-14.66) | 1.18 (1.14-1.23) |
| 2018/3/14 | 64.8 | 76.48 | 11.68 (8.85-14.54) | 1.18 (1.13-1.23) |
| 2018/3/15 | 65.57 | 77.17 | 11.6 (8.76-14.48) | 1.18 (1.13-1.22) |
| 2018/3/16 | 66.19 | 77.87 | 11.68 (8.83-14.58) | 1.18 (1.13-1.22) |
| 2018/3/17 | 66.69 | 78.41 | 11.72 (8.85-14.62) | 1.18 (1.13-1.22) |
| 2018/3/18 | 67.16 | 78.88 | 11.72 (8.84-14.63) | 1.17 (1.13-1.22) |
| 2018/3/19 | 67.89 | 79.37 | 11.49 (8.6-14.41) | 1.17 (1.13-1.21) |
| 2018/3/20 | 68.51 | 79.92 | 11.41 (8.52-14.35) | 1.17 (1.12-1.21) |
| 2018/3/21 | 69.01 | 80.66 | 11.66 (8.75-14.61) | 1.17 (1.13-1.21) |
| 2018/3/22 | 69.49 | 81.12 | 11.62 (8.7-14.58) | 1.17 (1.12-1.21) |
| 2018/3/23 | 70.01 | 81.46 | 11.45 (8.52-14.41) | 1.16 (1.12-1.21) |
| 2018/3/24 | 70.48 | 81.73 | 11.26 (8.32-14.22) | 1.16 (1.12-1.2) |
| 2018/3/25 | 70.87 | 81.98 | 11.11 (8.17-14.09) | 1.16 (1.11-1.2) |
| 2018/3/26 | 71.42 | 82.43 | 11.01 (8.06-13.99) | 1.15 (1.11-1.2) |
| 2018/3/27 | 71.91 | 82.84 | 10.93 (7.98-13.93) | 1.15 (1.11-1.2) |
| 2018/3/28 | 72.41 | 83.17 | 10.76 (7.8-13.76) | 1.15 (1.11-1.19) |
| 2018/3/29 | 72.94 | 83.54 | 10.6 (7.63-13.61) | 1.15 (1.1-1.19) |
| 2018/3/30 | 73.36 | 83.85 | 10.49 (7.51-13.5) | 1.14 (1.1-1.19) |
| 2018/3/31 | 73.62 | 84.03 | 10.41 (7.43-13.43) | 1.14 (1.1-1.18) |
| 2018/4/1 | 73.97 | 84.22 | 10.25 (7.26-13.27) | 1.14 (1.1-1.18) |
| 2018/4/2 | 74.38 | 84.54 | 10.17 (7.18-13.2) | 1.14 (1.1-1.18) |
| 2018/4/3 | 74.73 | 84.79 | 10.07 (7.07-13.1) | 1.13 (1.09-1.18) |
| 2018/4/4 | 75.05 | 85.06 | 10.01 (7.01-13.04) | 1.13 (1.09-1.18) |
| 2018/4/5 | 75.35 | 85.24 | 9.89 (6.89-12.93) | 1.13 (1.09-1.17) |
| 2018/4/6 | 75.6 | 85.39 | 9.78 (6.77-12.83) | 1.13 (1.09-1.17) |
| 2018/4/7 | 75.77 | 85.57 | 9.8 (6.79-12.85) | 1.13 (1.09-1.17) |
| 2018/4/8 | 76.02 | 85.73 | 9.72 (6.7-12.77) | 1.13 (1.09-1.17) |
| 2018/4/9 | 76.3 | 85.88 | 9.58 (6.56-12.63) | 1.13 (1.09-1.17) |
| 2018/4/10 | 76.46 | 86.02 | 9.56 (6.54-12.62) | 1.13 (1.08-1.17) |
| 2018/4/11 | 76.62 | 86.15 | 9.53 (6.5-12.59) | 1.12 (1.08-1.17) |
| 2018/4/12 | 76.73 | 86.31 | 9.58 (6.55-12.64) | 1.12 (1.08-1.17) |
| 2018/4/13 | 76.87 | 86.47 | 9.61 (6.58-12.67) | 1.12 (1.08-1.17) |
| 2018/4/14 | 76.95 | 86.56 | 9.61 (6.57-12.67) | 1.12 (1.08-1.17) |
| 2018/4/15 | 77.03 | 86.62 | 9.59 (6.56-12.66) | 1.12 (1.08-1.17) |
| 2018/4/16 | 77.08 | 86.74 | 9.66 (6.63-12.73) | 1.13 (1.09-1.17) |
| 2018/4/17 | 77.15 | 86.8 | 9.66 (6.62-12.73) | 1.13 (1.08-1.17) |
| 2018/4/18 | 77.25 | 86.84 | 9.59 (6.56-12.66) | 1.12 (1.08-1.17) |
| 2018/4/19 | 77.3 | 86.93 | 9.63 (6.59-12.7) | 1.12 (1.08-1.17) |
| 2018/4/20 | 77.35 | 86.93 | 9.58 (6.54-12.65) | 1.12 (1.08-1.17) |
| 2018/4/21 | 77.4 | 86.97 | 9.57 (6.53-12.64) | 1.12 (1.08-1.17) |
| 2018/4/22 | 77.45 | 87.01 | 9.56 (6.52-12.64) | 1.12 (1.08-1.16) |
| 2018/4/23 | 77.47 | 87.03 | 9.56 (6.52-12.63) | 1.12 (1.08-1.16) |
| 2018/4/24 | 77.49 | 87.05 | 9.56 (6.52-12.64) | 1.12 (1.08-1.16) |
| 2018/4/25 | 77.5 | 87.07 | 9.57 (6.53-12.65) | 1.12 (1.08-1.16) |
| 2018/4/26 | 77.54 | 87.09 | 9.55 (6.51-12.63) | 1.12 (1.08-1.16) |
| 2018/4/27 | 77.54 | 87.11 | 9.58 (6.53-12.65) | 1.12 (1.08-1.16) |
| 2018/4/28 | 77.55 | 87.13 | 9.58 (6.54-12.66) | 1.12 (1.08-1.16) |
| 2018/4/29 | 77.59 | 87.13 | 9.54 (6.5-12.62) | 1.12 (1.08-1.16) |
| 2018/4/30 | 77.59 | 87.15 | 9.56 (6.52-12.64) | 1.12 (1.08-1.16) |
| 2018/5/1 | 77.6 | 87.15 | 9.55 (6.51-12.63) | 1.12 (1.08-1.16) |
| 2018/5/2 | 77.61 | 87.17 | 9.56 (6.52-12.64) | 1.12 (1.08-1.16) |
| 2018/5/3 | 77.63 | 87.19 | 9.57 (6.53-12.65) | 1.12 (1.08-1.16) |
| 2018/5/4 | 77.64 | 87.19 | 9.55 (6.51-12.63) | 1.12 (1.08-1.16) |
| 2018/5/5 | 77.65 | 87.19 | 9.54 (6.5-12.62) | 1.12 (1.08-1.16) |
| 2018/5/6 | 77.66 | 87.19 | 9.54 (6.49-12.61) | 1.12 (1.08-1.16) |
| 2018/5/7 | 77.68 | 87.21 | 9.54 (6.5-12.62) | 1.12 (1.08-1.16) |
| 2018/5/8 | 77.68 | 87.21 | 9.54 (6.5-12.62) | 1.12 (1.08-1.16) |
| 2018/5/9 | 77.71 | 87.25 | 9.55 (6.5-12.63) | 1.12 (1.08-1.16) |
| 2018/5/10 | 77.71 | 87.25 | 9.55 (6.5-12.63) | 1.12 (1.08-1.16) |
| 2018/5/11 | 77.72 | 87.25 | 9.54 (6.5-12.62) | 1.12 (1.08-1.16) |
| 2018/5/12 | 77.73 | 87.25 | 9.52 (6.48-12.6) | 1.12 (1.08-1.16) |
| 2018/5/13 | 77.75 | 87.25 | 9.51 (6.46-12.59) | 1.12 (1.08-1.16) |
| 2018/5/14 | 77.78 | 87.25 | 9.48 (6.43-12.55) | 1.12 (1.08-1.16) |
| 2018/5/15 | 77.78 | 87.25 | 9.48 (6.43-12.55) | 1.12 (1.08-1.16) |
| 2018/5/16 | 77.79 | 87.25 | 9.47 (6.42-12.55) | 1.12 (1.08-1.16) |
| 2018/5/17 | 77.82 | 87.27 | 9.46 (6.41-12.54) | 1.12 (1.08-1.16) |
| 2018/5/18 | 77.82 | 87.27 | 9.46 (6.41-12.54) | 1.12 (1.08-1.16) |
| 2018/5/19 | 77.85 | 87.27 | 9.42 (6.38-12.5) | 1.12 (1.08-1.16) |
| 2018/5/20 | 77.86 | 87.27 | 9.41 (6.37-12.49) | 1.12 (1.08-1.16) |

† Cumulative cases of influenza-related hospitalizations per 100,000 inhabitants

Ω Compared with non-carnival region

₤ Carnival period

Abbreviation: CI, confidence interval.

**Table S5. Increase of COVID-19 cases per 100,000 inhabitants in the Netherlands**

| Day | Cumulative Cases^†^ | | Absolute rate difference^Ω^  (95% CI) | Rate ratio^Ω^  (95% CI) |
| --- | --- | --- | --- | --- |
|  | Non-carnival region | Carnival region |  |  |
| 2020/2/23^₤^ | - | - | - | - |
| 2020/2/24^₤^ | - | - | - | - |
| 2020/2/25^₤^ | - | - | - | - |
| 2020/2/26 | - | - | - | - |
| 2020/2/27 | 0 | 0.02 | 0.02 (-0.01-0.11) | 13.18 (0.13-Inf) |
| 2020/2/28 | 0.01 | 0.02 | 0.01 (-0.03-0.1) | 2.53 (0.05-134.09) |
| 2020/2/29 | 0.02 | 0.04 | 0.02 (-0.04-0.12) | 1.74 (0.17-12.81) |
| 2020/3/1 | 0.03 | 0.06 | 0.03 (-0.04-0.15) | 1.93 (0.31-10.18) |
| 2020/3/2 | 0.03 | 0.06 | 0.03 (-0.04-0.15) | 1.93 (0.31-10.18) |
| 2020/3/3 | 0.09 | 0.25 | 0.16 (0.02-0.34) | 2.76 (1.15-6.71) |
| 2020/3/4 | 0.16 | 0.35 | 0.19 (0.02-0.4) | 2.16 (1.08-4.26) |
| 2020/3/5 | 0.4 | 0.55 | 0.15 (-0.08-0.42) | 1.37 (0.83-2.21) |
| 2020/3/6 | 0.62 | 0.96 | 0.34 (0.04-0.68) | 1.55 (1.06-2.24) |
| 2020/3/7 | 0.86 | 1.57 | 0.72 (0.34-1.14) | 1.84 (1.36-2.48) |
| 2020/3/8 | 1.07 | 2.51 | 1.44 (0.97-1.96) | 2.34 (1.82-3.01) |
| 2020/3/9 | 1.16 | 3.56 | 2.4 (1.85-2.99) | 3.06 (2.45-3.84) |
| 2020/3/10 | 1.4 | 4.21 | 2.82 (2.22-3.46) | 3.02 (2.46-3.71) |
| 2020/3/11 | 1.69 | 5.83 | 4.13 (3.43-4.88) | 3.44 (2.87-4.12) |
| 2020/3/12 | 2.11 | 7.03 | 4.93 (4.15-5.75) | 3.34 (2.84-3.93) |
| 2020/3/13 | 2.6 | 9.28 | 6.69 (5.8-7.62) | 3.57 (3.09-4.13) |
| 2020/3/14 | 3.07 | 11.08 | 8.02 (7.05-9.03) | 3.61 (3.17-4.13) |
| 2020/3/15 | 3.67 | 12.7 | 9.03 (7.98-10.11) | 3.46 (3.06-3.91) |
| 2020/3/16 | 4.69 | 15.58 | 10.89 (9.73-12.1) | 3.32 (2.98-3.71) |
| 2020/3/17 | 5.87 | 18.52 | 12.66 (11.39-13.97) | 3.16 (2.86-3.48) |
| 2020/3/18 | 7.13 | 22.14 | 15.01 (13.62-16.44) | 3.1 (2.84-3.39) |
| 2020/3/19 | 9 | 25.58 | 16.58 (15.08-18.13) | 2.84 (2.62-3.08) |
| 2020/3/20 | 11.37 | 30.18 | 18.81 (17.17-20.49) | 2.65 (2.47-2.86) |
| 2020/3/21 | 14.2 | 35.49 | 21.29 (19.51-23.12) | 2.5 (2.34-2.67) |
| 2020/3/22 | 16.44 | 41.2 | 24.76 (22.84-26.73) | 2.51 (2.36-2.67) |
| 2020/3/23 | 18.76 | 45.86 | 27.1 (25.06-29.18) | 2.44 (2.31-2.59) |
| 2020/3/24 | 21.95 | 53.98 | 32.03 (29.82-34.28) | 2.46 (2.33-2.59) |
| 2020/3/25 | 26.1 | 60.62 | 34.53 (32.17-36.92) | 2.32 (2.21-2.44) |
| 2020/3/26 | 30.89 | 68.76 | 37.87 (35.36-40.42) | 2.23 (2.12-2.33) |
| 2020/3/27 | 36.19 | 78.78 | 42.59 (39.89-45.32) | 2.18 (2.08-2.27) |
| 2020/3/28 | 41.81 | 87.94 | 46.13 (43.27-49.02) | 2.1 (2.02-2.19) |
| 2020/3/29 | 46.83 | 97.28 | 50.45 (47.45-53.5) | 2.08 (2-2.16) |
| 2020/3/30 | 50.87 | 105.01 | 54.14 (51.01-57.3) | 2.06 (1.99-2.14) |

† Cumulative cases of COVID-19 cases per 100,000 inhabitants

Ω Compared with non-carnival region

₤ Carnival period

Abbreviation: CI, confidence interval.

**Supplemental Figures**

**Figure S1. Daily distribution of cumulative cases of influenza-related hospitalizations during the 2017/2018 influenza epidemic in the Netherlands**


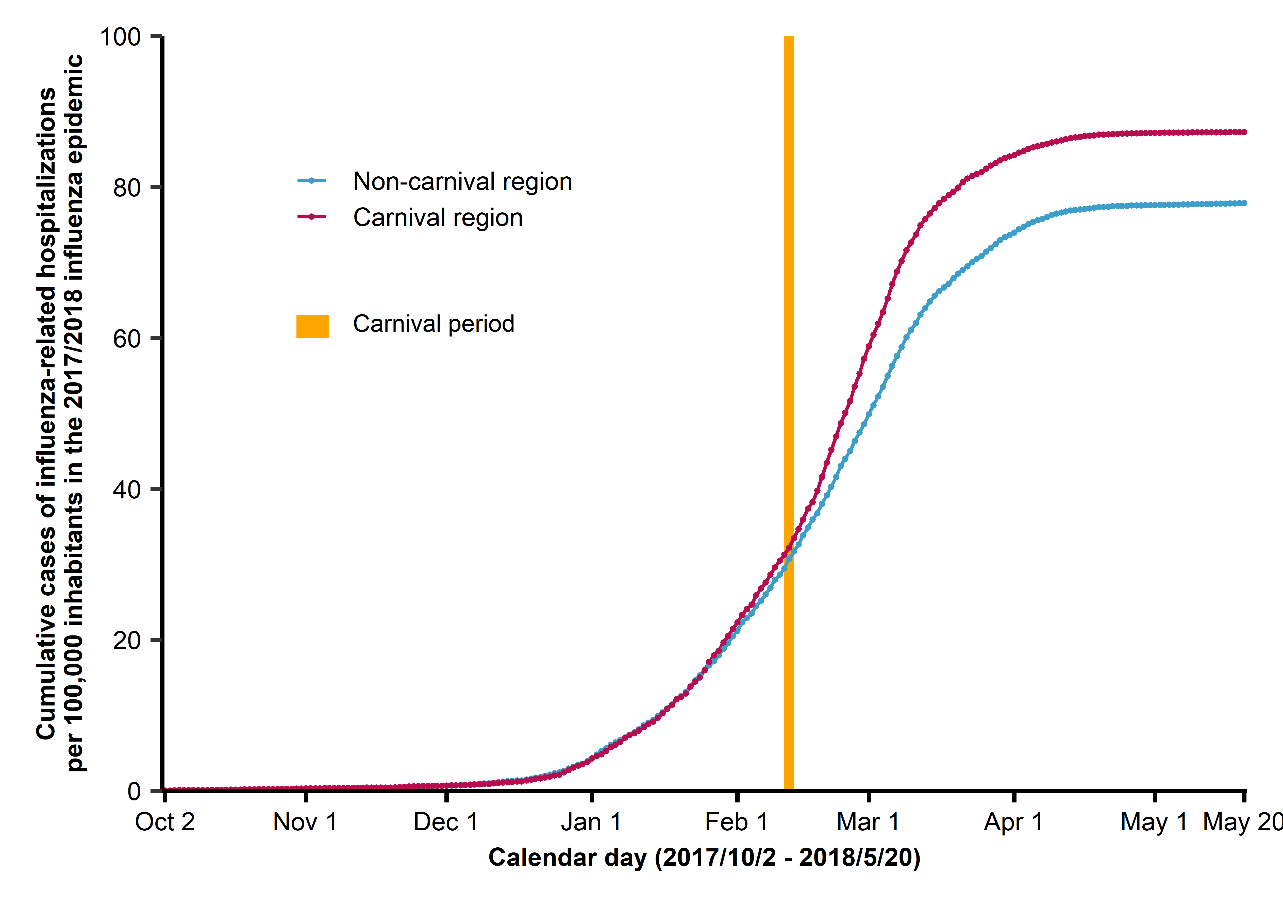


**Figure S2. Daily distribution of cumulative cases of COVID-19 in the Netherlands**


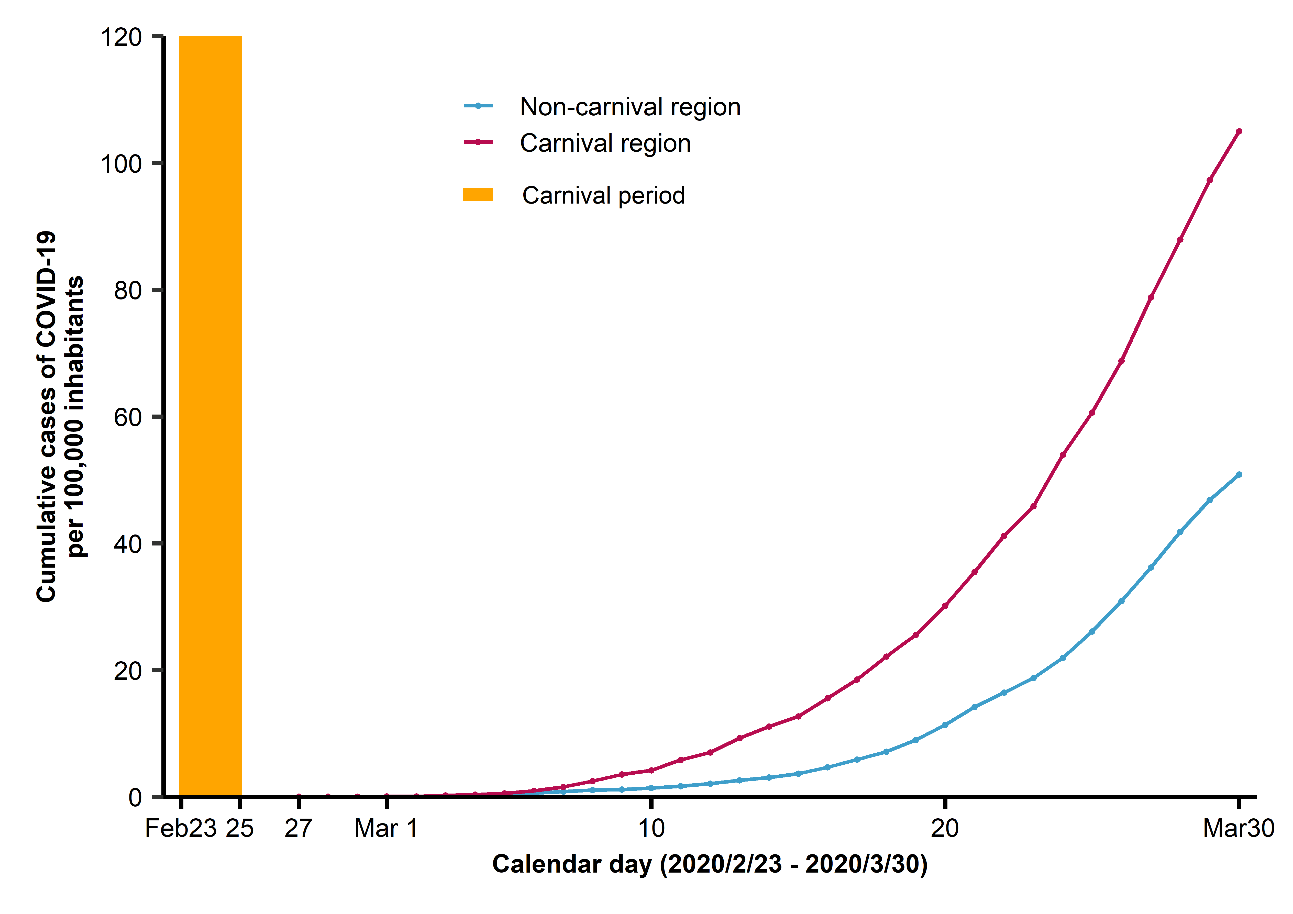

Supplement: Supplementary file 1 — Additional file 1: Table S1. Categorization of carnival region and non-carnival region at municipal level in 2018. Table S2. Categorization of carnival region and non-carnival region at municipal level in 2019. Table S3. Comparison of regional statistics between non-carnival region and carnival region in 2019. Table S4. Influenza-related hospitalizations per 100,000 inhabitants in the 2017/2018 influenza epidemic in the Netherlands. Table S5. Increase of COVID-19 cases per 100,000 inhabitants in the Netherlands. Figure S1. Daily distribution of cumulative cases of influenza-related hospitalizations during the 2017/2018 influenza epidemic in the Netherlands. Figure S2. Daily distribution of cumulative cases of COVID-19 in the Netherlands. [file 12889_2020_9612_MOESM1_ESM.docx]
